# Review of "System Modeling in Cellular Biology: From Concepts to Nuts and Bolts" by Szallasi, Stelling and Periwal

**DOI:** 10.1186/1475-925X-6-4

**Published:** 2007-01-30

**Authors:** Patrick E McSharry

**Affiliations:** 1 Department of Engineering Science, University of Oxford, Parks Road, Oxford OX1 3PJ, UK; 2 Oxford Centre for Integrative Systems Biology, University of Oxford, Oxford OX1 3QU, UK

## Abstract

"System Modeling in Cellular Biology: From Concepts to Nuts and Bolts" by Szallasi, Stelling and Periwal introduces the relevant concepts, terminology, and techniques of this field of science. It emphasises the modelling and computational challenges of taking a multidisciplinary approach to biology. This book provides a comprehensive introduction to systems biology and will form a valuable resource for students, teachers and researchers from both experimental and theoretical disciplines.

Systems biology, a multidisciplinary field of research, is suddenly gaining new attention from scientists in both academia and industry. It involves the construction of quantitatively predictive models of biological systems with the aim of understanding, predicting and controlling physiological behaviour by integrating knowledge of interactions at molecular, cellular and population levels. This book draws together contributions from writers within different disciplines and demonstrates the value of bringing together theorists from computer science, engineering, physics, mathematics and statistics with experimentalists from biochemistry, chemistry, biology and physiology.

This collection of chapters gives a thorough introduction to systems biology, providing a clearly structured list of reasons why such a systems approach is required in biology. By using well chosen examples and illustrative applications, the book manages to convey some of the complicated concepts underpinning research in systems biology. It explains why this approach relies on an iterative cycle between theory, modelling and experiments using high-throughput techniques.

The book emphasises the diverse range of skills required to successfully contribute to research in systems modelling and calls for a coordinated multidisciplinary approach where experimentalists and theorists collaborate and share resources. It clearly demonstrates that this collaboration should go beyond the sharing of experimental datasets and models if the results of research in systems biology are to be used to understand biological systems at the predictive level needed for the diagnosis, prevention and treatment of medical disease. The growth of multidisciplinary doctoral training centres and recent availability of funding to encourage research in systems biology has the potential to deliver researchers with sufficient knowledge of both experimental and theoretical issues. The authors of this book are well known for their contributions to the field of systems biology and the integration of their extensive experience is perhaps the best way to form a comprehensive introduction to the advantages, disadvantages and challenges of systems modelling applied to cellular biology.

The four principle sections of the book are structured around general concepts, modelling approaches, models and reality and computational modelling. A clear account of the role of modelling in systems biology is provided, highlighting the philosophical arguments behind model selection and summarising the different kinds of modelling approaches available. It also describes important concepts such as nonlinearity, feedback loops, sensitivity analysis, parameter exploration and model consistency. By focusing on the interplay between complexity and robustness, it emphasises the importance of modelling the network structure and discusses concepts such as stability, fragility and evolvability. It also presents the advantages and disadvantages of modules when modelling complex systems. The book then proceeds to describe the wide range of possible modelling approaches; these include Bayesian inference, constraint-based models, nonlinear ordinary differential equations, qualitative models, stochastic models and spatially extended models. One of the book's strengths is that relevant applications and examples are presented throughout to help convey the essential concepts underlying each modelling approach. The applications include metabolic networks, molecular interaction networks, genetic regulatory networks, intracellular kinetics and reaction-diffusion systems. The next section describes the range of experimental data available and emphasises the many sources of observational uncertainty and measurement error that should be accounted for when modelling. It covers issues such as data acquisition, methods for identifying architecture and dynamics from experimental data, applications of control theory, synthetic gene regulatory systems and multilevel modelling. The book closes with a description of the computational techniques required when implementing a systems modelling approach. By discussing computational constraints, numerical simulation methods and software infrastructure, it is apparent that knowing the limitations and cost of increasing model size is important for both experimentalists and modellers.

"Systems biology in cellular biology" provides a great resource for researchers from different backgrounds who wish to contribute to systems biology. The notable collection of contributors helps to provide a comprehensive view of the latest developments in the exciting new field of systems biology. The book forms an impressive overview of the entire field which is suitable for students, teachers and researchers, whether they be experimentalists or theorists.

